# *Byrsonima fagifolia* Niedenzu Apolar Compounds with Antitubercular Activity

**DOI:** 10.1093/ecam/nen077

**Published:** 2011-06-15

**Authors:** C. T. Higuchi, M. Sannomiya, F. R. Pavan, S. R. A. Leite, D. N. Sato, S. G. Franzblau, L. V. S. Sacramento, W. Vilegas, C. Q. F. Leite

**Affiliations:** ^1^Unesp, Universidade Estadual Paulista, Faculdade de Ciências Farmacêuticas, Rodovia Araraquara-Jaú km 01, CEP 14801-902, Araraquara–SP, Brazil; ^2^Unesp, Universidade Estadual Paulista, Instituto de Química, Rua Francisco Degni s/n. Bairro Quitandinha, C.P.335. CEP 14800-900, Araraquara–SP, Brazil; ^3^Instituto Adolfo Lutz, Laboratório de Micobacteriologia, Rua Minas, 887, CEP 14085-410, Ribeirão Preto, SP, Brazil; ^4^Institute for Tuberculosis Research, College of Pharmacy, University of Illinois at Chicago, USA

## Abstract

Bioassay-guided fractionation of the chloroform extract of *Byrsonima fagifolia* leaves led to the isolation of active antitubercular compounds alkane dotriacontane (Minimal Inhibitory Concentration—MIC, 62.5 **μ**g mL^−1^), triterpenoids as bassic acid (MIC = 2.5 **μ**g mL^−1^), **α**-amyrin acetate (MIC = 62.5 **μ**g mL^−1^), a mixture of lupeol, **α**- and **β**-amyrin (MIC = 31.5 **μ**g mL^−1^) and a mixture of lupeol, and acetates of **α**- and **β**-amyrin (MIC = 31.5 **μ**g mL^−1^). The antimycobacterial activity was determined by the Microplate Alamar Blue Assay (MABA) and the structures of promising compounds were determined by spectroscopic analysis. This investigation constitutes the first report of a chemical and antitubercular study of apolar compounds from *B. fagifolia* Niedenzu (IK).

## 1. Introduction

*Mycobacterium tuberculosis*, the agent of tuberculosis, is responsible for high mortality worldwide, killing roughly 1.7 million people annually [1]. The situation with multidrug resistant (MDR) tuberculosis (TB) today worries the health authorities of the whole world, mainly in the developing countries, where the situation is more severe. The appearance of resistant strains to the medicines now in use makes urgent the search for new synthetic or natural tuberculostatic drugs. There are several reasons that justify the need to search for new drugs for TB, for example, improvement of current treatment by shortening its duration, to get efficient treatment for MDR TB and to eradicate the latent infection. So, the development of new drugs for shortening the duration of the treatment and to fight against multidrug resistant tuberculosis strains is urgent [2].

An approach to the search for new drugs is to look in nature, mainly for the extremely rich and varied flora of the tropical areas. In this search, information obtained from folk knowledge and traditional medicine of different cultures can be valuable. This trend in associating popular or alternative medicine with pharmaceutical research has been growing for some years [3].

*Byrsonima fagifolia* Niedenzu (IK) is a member of the Malpighiaceae family and is a native species from the Brazilian Cerrado (savannah-like vegetation). The Cerrado is considered the most extensive woodland-savannah in South America and contains over 5000 species of higher plants [4], some of then with antimicrobial activities [5]. *B. fagifolia* is popularly known as murici-cascudo or murici-vermelho [6]. In Brazilian folk medicine, the leaves of *B. fagifolia* are used as an antiemetic, diuretic, febrifuge and to treat peptic ulcers [6]. Previously, we also have reported the antiulcer activity of *B. crassa* extracts [7] and the antidiarrhoeal activity observed with the methanol extract of *B. cinera* [8]. Despite the popular use of *B. fagifolia* as a medicinal plant, there are no data on the antitubercular activity of its leaves extract or compounds.

Plants extracts are attractive sources of new drugs, and bioassay-guided fractionation is the state-of-art process to identify the active compounds contained in crude natural products. The aim of this study is to evaluate by Microplate Alamar Blue Assay (MABA), the potential antitubercular activity of *B. fagifolia* leaves extracts, enriched fractions and pure compounds, identified by phytochemical analysis.

## 2. Methods

### 2.1. Plant Material

The fresh leaves of *B. fagifolia* Niedenzu (IK) were collected at Estrada do Brejinho de Nazaré, Tocantins State, Brazil (11°01′S, 48°34′W, elevation 240 m) and the species was identified by Dr Eduardo Ribeiro dos Santos of Tocantins University. A voucher specimen was deposited in the herbarium of the same university under the number 6398.

### 2.2. Extraction and Isolation

The air-dried and powdered leaves (2.0 kg) of *B. fagifolia* were extracted exhaustively with chloroform, methanol and 80% methanol (methanol/water 80/20 v/v), successively at room temperature (48 h for each solvent). Solvents were evaporated at 60°C under reduced pressure to yield the chloroform (92.7 g), methanol (303.8 g) and 80% methanol (201.2 g) extracts. The yields (w/w) for the extracts from the dried powders of *B. fagifolia* leaves were 4.63, 15.19 and 10.06%, respectively.

A portion of the chloroform extract (10.0 g) was chromatographed on a Merck silica gel column (15 cm × 6.0 i.d) in order to separate the compounds according to their polarity.

The column was eluated sequentially with hexane, then dichloromethane and finally methanol. Evaporation of the solvents yielded the dry eluates from hexane (0.76 g), dichloromethane (3.9 g) and methanol (2.2 g).

The fraction eluated by hexane (0.76 g) was rechromatographied on a silica gel 60 column (13.0 × 2.0 cm i.d.) and eluated with pure *n*-hexane. Analogously, the other two fractions (eluated with methanol and dichloromethane) were fractionated on a similar column (13.0 × 3.0 cm i.d.) using pure chloroform as mobile phase, and gradually increasing the polarity with methanol (for the first fraction) or dichloromethane (for the second one).

### 2.3. Gas Chromatography Analysis

Gas chromatography (GC) analysis for hydrocarbon identification was performed using a Varian 3380 gas chromatograph equipped with a fused silica CBP-5 capillary column (25 m × 0.33 mm i.d.; film thickness 0.5 m) and a flame ionization detector (FID). Hydrogen was used as the carrier gas (60 kPa), and the injection split ratio was 1 : 30. The injection temperature was 250°C; the column temperature was held at 50°C for 1 min, and then increased to 300°C at 10°C min^−1^, and this temperature was held for 10 min; the detector temperature was 280°C. Samples of 1 *μ*lLwere injected using a 10 *μ*L Hamilton syringe. The fraction eluated by hexane from the silica gel column of the *B. fagifolia* extract in chloroform was then analyzed by GC, and the chromatograms compared with standard hydrocarbons (straight chain alkanes kit, C_9_ to C_32_, from Aldrich), obtaining the retention times.

### 2.4. Structural Identification of the Triterpenes

Structural identification of the triterpenes was performed by ^13^C Nuclear Magnetic Resonance (NMR) spectroscopy. The NMR spectra in deuterated chloroform (CDCl_3_) were obtained using a Varian INOVA 500 spectrometer, operating at 500 MHz for ^1^H and 150 MHz for ^13^C. Chemical shifts were given in **δ** (p.p.m.) using tetramethylsilane (TMS) as the internal standard. The NMR spectra data obtained were compared with those reported in the literature [9]. The identification of the isomeric amyrins and its acetates (*α* and *β*) is perfectly possible by the ^13^C NMR spectrum, even if they are mixed, once some similar bands of the isomeric ones have very different chemical shifts. Similarly, other triterpenes may be identified in mixtures, without previous separation [10].

### 2.5. Antitubercular Activity Assay

The antitubercular activity of chloroform, methanol and 80% methanol extracts of *B. fagifolia* leaves, the enriched fractions and pure compounds were determined using the MABA [11] as the analytical method. Stock solutions of the tested compounds were prepared in dimethyl sulfoxide [11] and were diluted in Middlebrook 7H9 (Difco) broth supplemented with oleic acid, albumin, dextrose and catalase (OADC enrichment—BBL/Becton-Dikinson, Sparks, MD, USA) to obtain final sample concentrations ranges of 0.15–1600 *μ*g mL^−1^. Isoniazid was solubilized with distilled water according to the manufacturers' recommendations (Difco laboratories, Detroit, MI, USA) and used as a positive control drug. *M. tuberculosis* H_37_Rv ATCC 27294 was grown for 7–10 days in Middlebrook 7H9 supplemented with OADC added of 0.05% Tween 80 to avoid clumps. Suspensions were prepared and their turbidities matched to a McFarland no. 1 (turbidity standard). After further dilution of 1 : 25 in Middlebrook 7H9 supplemented with OADC, the inoculum was added to each well of the 96 well microtiter plate (Falcon 3072; Becton–Dickinson, Lincoln Park, NJ, USA) together with the compounds. Samples were set up in triplicate. Cultures were in incubated for 7 days at 37°C, and after additioned Alamar Blue for the reading. The minimum inhibitory concentration (MIC) was defined as the lowest concentration resulting in 90% inhibition of growth of *M. tuberculosis* [11] measuring the fluorescence (excitation/emission of 530/590 filters, resp.) in a SPECTRAfluor Plus (Tecan) [12]. For standard test, the MIC value of isoniazid was determined each time. The acceptable MIC of Isoniazid ranged from 0.015 to 0.05 *μ*g mL^−1^.

## 3. Results

**Figure 1:**
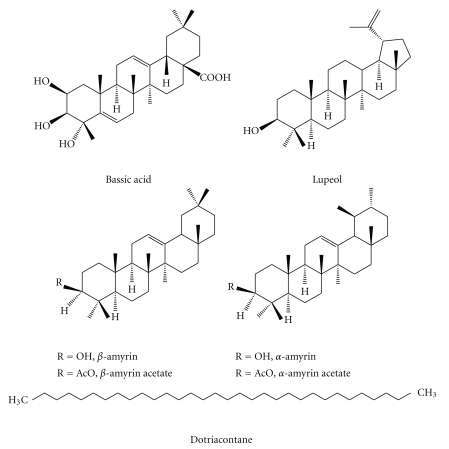
Compounds identified from the chloroform extract of *Byrsonima fagifolia*.

The fractions originating from the initial chromatography of the extract in chloroform of *B. fagifolia* leaves were submitted for subsequent column chromatographic separations, as described in Experimental section, yielded the following compounds:

The fraction eluated with hexane (0.76 g) yielded 530 mg of the *n*-alkane dotriacontane and 15.4 mg of the triterpene *α*-amyrin acetate. The triterpene bassic acid (234 mg) was obtained from 2.0 g of the methanol eluate and the dichloromethane fraction (2.0 g) resulted in a mixture of the triterpenes lupeol, *α*- and *β*-amyrin (223 mg), as well as the corresponding acetates (124 mg). The structures of the compounds are shown in Figure 1. The alkane dotriacontane was identified by GC and all triterpenes were identified by their ^13^C NMR spectra.

The chloroform, methanol and 80% methanol extracts of leaves yielded MIC values of 62.5, 250 and 500 *μ*g mL^−1^, respectively (Table 1). MIC of 31.25 *μ*g mL^−1^ was observed in both mixtures obtained from the dichloromethane fraction of the chloroform extract (one with lupeol, *α*- and *β*-amyrin and the other with lupeol and acetates of *α*- and *β*-amyrin). The pure compounds *α*-amyrin acetate and dotriacontane, both showed MIC of 62.5 *μ*g mL^−1^, and the bassic acid presented a very promising MIC value of 2.5 *μ*g mL^−1^. The MIC of Isoniazid, used as a positive control drug, was 0.03 *μ*g mL^−1^.

## 4. Discussion

According to Copp [13], terpenes dominate the number of natural products reported with antimycobacterial activity. Several terpenes (diterpenes, sesquiterpenes, sesterpenes and triterpenes) have demonstrated this biological activity [14].

Tosun et al. [15] considered inactive those plants extracts that could not prevent growth of *M. tuberculosis* up to a concentration of 200 *μ*g mL^−1^ and according to Gu et al. [16] the MIC value of ≤128 *μ*g mL^−1^ is defined as active. In the present study, the mixture of lupeol, *α*- and *β*-amyrin displayed lower MIC (31.25 *μ*g mL^−1^) than those previously reported with pure lupeol [17] and *α*- and *β*-amyrin [18] isolated from *Chuquiraga ulcina* H. et A. and from *Asteraceae* Martinov flowers (64 *μ*g mL^−1^). Their synergistic activity in the mixture might explain these results. In theory, fractionation allows for the isolation of pure compounds with higher activity than the mixture, but it is common the original extract or the mixture to have better activity. Houghton et al. [19] published an excellent review about this matter. The mixture containing lupeol and *α*- and *β*-amyrin acetates showed the same MIC value of 31.25 *μ*g mL^−1^, suggesting that the acetylation of *α*- and *β*-amyrin does not influence their activity. A MIC of 62.5 *μ*g mL^−1^, for the pure acetate of *α*-amyrin, double the value for the mixture, reinforces the synergistic effect among the components of the mixture against *M. tuberculosis*.

In previous study performed in Brazil, the new triterpene lupenone was isolated from *B. microphyla* A. Juss. [20] an other species of *Byrsonima.* Lupenone was not found in *B. fagifolia* but its activity against *M. tuberculosis* was tested by our group being found MIC of 125 *μ*g mL^−1^ (Nasser ALM et al., unpublished data).

The triterpene bassic acid showed strong antitubercular activity with MIC values of 2.5 *μ*g mL^−1^. This value is comparable with that found by Woldemichael et al. [21] for diterpenes isolated from *Calceolaria pinnifolia* Cav. and sterols from *Ruprechitia triflora* Griseb. with MICs of 2–4 *μ*g mL^−1^. Although this MIC value is larger than the reference drug Isoniazid (MIC = 0.03 *μ*g mL^−1^), the inhibitory concentration value of 2.5 *μ*g mL^−1^ is comparable to MICs of the other first-line tuberculosis drugs, such as ethambutol (1–5 *μ*g mL^−1^) and streptomycin (2–8 *μ*g mL^−1^), and better than pyrazinamide (20–100 *μ*g mL^−1^) [22].

The biological activity against *Leishmania* [23] and the hypoglycemic activity [24] were already described for bassic acid but never for *M. tuberculosis*.

Dotriacontane is an alkane with 32 carbons, and its antitubercular activity (62.5 *μ*g mL^−1^) was also never described before, but the antimycobacterial activity of other alkanes is documented in an extensive review covering 12 years of natural products literature [16]. Two alkenes derived from the microbial products, cerulenin and thiolactomycin, inhibit the function of *β*-ketoacyl-acyl carrier protein (ACP) synthases, which are key regulators of type II fatty acid biosynthesis [25]. As mycolic acids are prevalent in the outer lipid layer of mycobacteria, inhibitors of fatty acid biosynthesis represent existing and potential antimycobacterial agents.

In conclusion, the bioassay-directed fractionation of the chloroform extract of *B. fagifolia* leaves yielded the pure triterpene bassic acid with potent anti-TB activity. Promising activities were also verified for the mixture of lupeol, *α*-amyrin and *β*-amyrin or their acetates, and for dotriacontane.

## Figures and Tables

**Table 1:** Determination of MIC values of extracts, fractions and compounds from leaves of *B. fagifolia* against *M. tuberculosis* ATCC 27294, using MABA.

Samples	MIC (*μ*g/mL)
Extracts	
80% MeOH	500
MeOH	250
CHCl_3_	62.5

Enriched fraction/compounds	
Mixture of lupeol, *α*- and *β*-amyrin	31.25
Mixture of lupeol, acetates of *α*- and *β*-amyrin	31.25
*α*-Amyrin acetate	62.5
Dotriacontane	62.5
Bassic acid	2.5

Reference drug	
Isoniazid	0.03

MeOH, Methanol extract; CHCl_3_, Chloroform extract.
